# *Helicobacter pylori* infection in infant rhesus macaque monkeys is associated with an altered lung and oral microbiome

**DOI:** 10.1038/s41598-024-59514-5

**Published:** 2024-05-01

**Authors:** Noah A. Siegel, Monica T. Jimenez, Clarissa Santos Rocha, Matthew Rolston, Satya Dandekar, Jay V. Solnick, Lisa A. Miller

**Affiliations:** 1 California National Primate Research Center, University of California Davis, Davis, CA USA; 2 Department of Medical Microbiology and Immunology, School of Medicine, University of California Davis, Davis, CA USA; 3 Department of Anatomy, Physiology and Cell Biology, School of Veterinary Medicine, University of California Davis, Davis, CA USA

**Keywords:** Paediatric research, Experimental models of disease, Microbiome

## Abstract

It is estimated that more than half of the world population has been infected with *Helicobacter pylori*. Most newly acquired *H. pylori* infections occur in children before 10 years of age. We hypothesized that early life *H. pylori* infection could influence the composition of the microbiome at mucosal sites distant to the stomach. To test this hypothesis, we utilized the infant rhesus macaque monkey as an animal model of natural *H. pylori* colonization to determine the impact of infection on the lung and oral microbiome during a window of postnatal development. From a cohort of 4–7 month-old monkeys, gastric biopsy cultures identified 44% of animals infected by *H. pylori*. 16S ribosomal RNA gene sequencing of lung washes and buccal swabs from animals showed distinct profiles for the lung and oral microbiome, independent of *H. pylori* infection. In order of relative abundance, the lung microbiome was dominated by the phyla Proteobacteria, Firmicutes, Bacteroidota, Fusobacteriota, Campilobacterota and Actinobacteriota while the oral microbiome was dominated by Proteobacteria, Firmicutes, Bacteroidota, and Fusobacteriota. In comparison to the oral cavity, the lung was composed of more genera and species that significantly differed by *H. pylori* status, with a total of 6 genera and species that were increased in *H. pylori* negative infant monkey lungs. Lung, but not plasma IL-8 concentration was also associated with gastric *H. pylori* load and lung microbial composition. We found the infant rhesus macaque monkey lung harbors a microbiome signature that is distinct from that of the oral cavity during postnatal development. Gastric *H. pylori* colonization and IL-8 protein were linked to the composition of microbial communities in the lung and oral cavity. Collectively, these findings provide insight into how *H. pylori* infection might contribute to the gut-lung axis during early childhood and modulate future respiratory health.

## Introduction

*Helicobacter pylori* is a common cause of chronic gastritis in humans and can elicit a range of disease phenotypes depending upon the host, bacterial virulence, and environmental factors (reviewed in^1^). It is estimated that half of the world’s population is infected with *H. pylori,* with prevalence varying by geographical location^2^. In the United States, a recent survey of veterans indicated a positive diagnosis of *H. pylori* infection in over 25% of patients^3^. Although colonization of the stomach imposes an increased risk of developing peptic ulcers (10–20%) and gastric cancer (1–2%), the majority of human subjects infected with *H. pylori* are asymptomatic^4^. *H. pylori* is primarily acquired in early childhood and unless the infected individual is treated with antibiotics, colonization persists for life^5–8^. Transmission of *H. pylori* is most likely through a buccal-gastro route, as the organism has been detected in both vomitus and buccal aspirates of infected children^9,10^.

When *H. pylori* is present in the stomach, it dominates the gastric microbial community, decreasing overall taxonomic diversity and comprising up to 97% of reads in culture-independent analysis^11^. As such, characterization of the human gastric microbiome has revealed two apparent phenotypes: one colonized with the gram-negative, microaerophilic bacteria *H. pylori* and one without. Experimental *H. pylori* infection also alters the microbial community structure and abundance at distant sites within the gastrointestinal tract in a murine model, although eradication treatment in humans has been reported to be successful in reverting to baseline alpha and beta diversity^12,13^. To date, it is unknown whether gastric *H. pylori* colonization can influence the lung microbiome on a short-term or persistent basis, but microaspiration may be a potential pathway for the direct introduction of the pathogen to the lung; *H. pylori* and associated virulence factors have been identified in the human adult lung^14–16^.

While bacteria pathogens can be aspirated into the respiratory tract, it remains unclear how the commensal population of the lung can be altered by the host beyond antibiotic treatments. Several reports have suggested a link between the community composition of the lung microbiome and respiratory diseases, including asthma, COPD, and cystic fibrosis^14,17–20^. However, many chronic diseases of the airways in adults are thought to be established in early childhood, which suggests a more thorough understanding of the development of the normal pediatric lung microbiome is critical^21^. A potential immunomodulatory role for *H. pylori* in the context of the gut-lung axis has been suggested by epidemiological findings in human populations demonstrating an inverse association between the development of childhood asthma and gastric colonization with *H. pylori*^22,23^. Correspondingly, introduction of *H. pylori* in a neonatal murine model has shown the induction of T regulatory cells via IL-18-producing dendritic cells is essential for immunosuppression of allergic airways disease development^24,25^.

Human microbiome studies have provided support for the notion that commensal bacteria are essential for maintaining physiological homeostasis and influencing disease development^26^. To determine whether the presence of gastric *H. pylori* infection can alter the composition of the lung and oral microbiome during infancy, we used the rhesus macaque monkey as a model of early childhood development. Rhesus monkeys in captive environments become naturally infected with an *H. pylori* strain virtually indistinguishable from strains identified in humans, and socially housed animals acquire *H. pylori* within the first year of life^27,28^. Here, we assessed the relationship between the gastric *H. pylori* infection status, lung microbiome, and oral microbiome in infant monkeys. We also evaluated whether the expression of IL-8 protein as a mucosal or systemic biomarker of *H. pylori* colonization is associated with microbial communities in either the lung or oral cavity.

## Methods

### Animals

Infant rhesus macaque (*Macaca mulatta*) monkeys aged 4–7 months (n = 25) were born at the California National Primate Research Center (CNPRC). All animals were socially housed, reared, and breastfed by dams indoors in an environment-controlled facility with an ambient temperature of 21–25 °C, relative humidity of 40–60%, and a 12 h light/dark cycle. All animals were provided water and commercial monkey chow ad libitum. Before, during, and after treatment, care and housing of animals complied with the Institute of Laboratory Animal Resources provisions and conforms to practices established by the Association for Assessment and Accreditation of Laboratory Animal Care International (AAALAC International). All animal procedures were approved by the University of California, Davis, Institutional Animal Care and Use Committee. All possible efforts were made to minimize pain and distress following the recommendations of the National Research Council publication, Guide for the Care and Use of Laboratory Animals, Eighth Edition.

### Biospecimen collection

Animals were fasted overnight before collection of blood, lung wash, buccal swab, and gastric biopsy. Animals were sedated with ketamine anesthesia (10 mg/kg) administered intramuscularly during sample collection. To avoid injury to the infant monkey respiratory tract, lung washes were obtained using a sterile single-use endotracheal tube and instillation of sterile PBS. PBS was instilled at a volume of 2 ml per kilogram body weight. A swab of the buccal cavity was collected prior to the lung wash, along with a blood sample for plasma collection. Endoscopy was conducted using a pediatric bronchoscope to collect gastric corpus biopsies for *H. pylori* culture as previously described^28^. Lung wash fluids, buccal swabs, and plasma was stored at -80 °C until used for analysis.

### *H. pylori* culture

Gastric biopsy specimens were placed in vials containing Brucella broth and transported to the laboratory for immediate processing as previously described^28^. All plates were incubated in an atmosphere of 5% O_2_, 7.6% CO_2_ for up to 8 days. *H. pylori* was identified conventionally by colony morphology (pinhead-sized translucent colonies), microscopy (gram-negative curved organisms), and biochemistry (oxidase, catalase, and urease positive). Colony forming units per gram of tissue specimen were calculated by enumerating colonies, adjusting for the dilution, and dividing by the tissue weight.

### Cytokine ELISA

IL-8 protein concentration in lung wash fluid and plasma was measured by ELISA DuoSet kit (R&D Systems, Minneapolis, MN) according to the manufacturer's instructions. The limit of detection for the IL-8 ELISA was 7.5 pg/mL.

### Demographics of animals evaluated for lung and oral microbiome composition

Demographics of animals evaluated by 16S rRNA gene sequencing analysis are listed in Supplemental Table 1. Of the 25 infant rhesus macaques (17 females, 8 males) initially screened for *H. pylori* infection, a total of 14 animals were selected for 16S rRNA gene sequencing analysis (6–7 months old, 13 females, 1 male). A predominantly female sample population was selected because of the potential for sex-dependent effects when evaluating the oral and lung microbiome, as observed by others in the oral microbiome^29^. Of the 14 animals evaluated for microbiome composition, 8 were *H. pylori*-negative and 6 were *H. pylori*-positive by gastric biopsy; the animals along with their dams were housed in four different rooms prior to biospecimen collection (Supplemental Table 1). Microbiome analysis of buccal swabs was limited to 12 out of 14 animals evaluated. Animals had a median weight within 0.1 kg and a median age within 0.8 months of each other.

**Table 1:** Demographics for study animals (n = 25).

	*H. pylori* ( +) (n = 11)	*H. pylori* (-)(n = 14)
Median *H. pylori* load (range)	5.9 cfu/g (3.8–8.1)	NA
Median age (range)	7.2 months (5.3–7.9)	6.6 months (4.5–7.6)
Median weight (range)	1.6 kg (1.2–1.7)	1.4 kg (1.0–1.7)
Weaning status (% weaned)	27	36
Median dam age (range)	96.2 months (89–103)	99.4 months (68–117)
Median dam weight (range)	7.1 kg (5.0–9.2)	7.7 kg (6.7–9.4)
Sex (% female)	55	71

### 16S rRNA gene amplicon sequencing library preparation

In brief, according to the manufacturer's instructions, DNA was isolated using the PowerSoil DNA Isolation Kit (Qiagen, USA). A nested PCR was used to amplify and barcode the V4 domain of the 16S ribosomal RNA (rRNA) gene. The first PCR was conducted using 1μL of DNA template and primers 27F (AGAGTTTGATCMTGGCTCAG) and 1492R (GGTTACCTTGTTACGACTT) were used to amplify a large portion of the 16S rRNA gene. The second PCR was conducted using 1μL of the resulting amplicon with the following primers to amplify the V4 domain:

515F: (**AATGATACGGCGACCACCGAGATCTACAC**NNNNNNNNTATGGTAATT*GT*GTGCCAGCMGCCGCGGTAA)

806R: (**CAAGCAGAAGACGGCATACGAGAT**NNNNNNNNAGTCAGTCAG*CC*GGACTACHVGGGTWTCTAAT).

Each primer used in the second PCR contained a unique 8 nt barcode (N) a primer pad (underlined), a two-base linker (italicized) and the Illumina adaptor sequences (bold text). A unique combination of these primers was used to barcode each sample PCR reaction contained 1 Unit Kapa2G Robust Hot Start Polymerase (Kapa Biosystems), 1X Kapa2G buffer with MgCl2, 10 mM NTPs and 10 pmol of each primer. The first PCR step cycling conditions were an initial incubation at 95 °C for 5 min, followed by 25 cycles of 95 °C for 30 s, 50 °C for 30 s, and 72 °C for 90 s and a final extension of 72 °C for 7 min. The second PCR conditions were an initial incubation at 95 °C for 3 min, followed by 25 cycles of 95 °C for 45 s, 50 °C for 60 s, and 72 °C for 90 s and a final extension of 72 °C for 10 min. The final PCR product was quantified on the Qubit instrument using the Qubit high-sensitivity DNA kit (Invitrogen) and individual amplicons were pooled together at the same concentration. Library quality was assessed using the Agilent Bioanalyzer 2100 High Sensitivity DNA assay.

### 16S rRNA gene amplicon library sequencing and bioinformatics

All samples were sequenced on an Illumina MiSeq platform at the Genome DNA Technology Core at the University of California, Davis. Demultiplexing of raw FASTQ files and adapter trimming of sequences was performed using dbcAmplicons version 0.8.5. (https://github.com/msettles/dbcAmplicons). The unmerged forward and reverse reads were imported into QIIME2^30^ version 2020.8 (https://docs.qiime2.org/2020.8/), and amplicon sequencing variants (ASVs) were determined following the DADA2 analysis pipeline^31^. Snakemake^32^ was used as a workflow manager to manage the QIIME2 environment (https://github.com/nasiegel88/tagseq-qiime2-snakemake-1). Each sequence was assigned to its given samples based on the given barcode. Reads that did not match any barcode were discarded (failed to meet minimum quality thresholds). Barcoded forward and reverse sequencing reads were quality filtered and merged. Sequences only observed once or in a single sample were also discarded. Chimeras were detected and filtered from paired end reads. A comparison of clustered sequences was performed against SILVA 138.

### Data processing, filtering, and trimming of reads

The data were filtered as follows: ambiguous phyla were removed, and phyla with a mean prevalence of less than 10 were removed. Taxa were agglomerated at the genus level if possible, and all taxa without genus-level taxonomic assignments were retained. Samples with less than 5000 reads were removed.

### Statistical analysis

All R packages used for analyses were installed in R 4.1.3 Studio software unless otherwise stated. Statistical analysis of microbial communities was performed primarily using the Bioconductor package Phyloseq (version 1.38.0). Spearman and Pearson correlations were done in R using the Microeco R package (version 0.7.0). Differential abundance analyses of filtered data were performed using Linear discriminant analysis Effect Size (LEfSe) as described in the literature^33,34^. Cytokine data were evaluated using ANOVA (1- or 2-way), unpaired t-test, or linear regression using rstatix (version 0.7.0). Outlier tests were performed where appropriate. Statistical significance was set at a *p* value < 0.05.

### Ethics approval

This study was performed in compliance with the National Institutes of Health Guide for the Care and Use of Laboratory Animals. Established policies of the Institutional Animal Care and Use Committee (IACUC) of the University of California, Davis were followed for biospecimen collections, housing, and medical care. This study was performed at the California National Primate Research Center (CNPRC), which is one of seven centers supported by the National Institutes of Health, Office of the Director (NIH/OD), and is accredited by the Association for the Assessment and Accreditation of Laboratory Animal Care International (AAALAC). The CNPRC maintains a breeding colony of rhesus macaque monkeys in outdoor corrals or indoor housing. The authors have complied with the ARRIVE guidelines^66^.

## Results

### The infant rhesus monkey lung and oral microbiomes have distinct profiles

*H. pylori* infection is highly prevalent in socially housed captive colonies of rhesus macaque monkeys^27,35^. We have previously detected *H. pylori* infection in infant rhesus monkeys as early as 12 weeks of age, with approximately 90% of animals becoming culture positive at one year of age^28^. To assess whether gastric bacterial infection is associated with an altered commensal microbiome for the respiratory tract, we first evaluated a cohort of 25 infant monkeys (4–7 months of age, 8 males and 17 females) by gastric biopsy, lung wash and blood collection (Table 1). From our initial pool of 25 study animals, a total of 11 infant monkeys (6 males and 5 females) were identified as culture positive for *H. pylori* from gastric biopsy samples; we did not find differences in age as well as housing status between *H. pylori* culture positive versus negative animals (Fig. S1A, B, Supplemental Table 1). There was an inverse relationship between infant *H. pylori* infection and maternal age, with younger dams associated with offspring having a higher *H. pylori* gastric load (*p* = 0.007) (Fig. S2).

To investigate whether gastric *H. pylori* infection can influence the composition of microbial communities in the lung, we conducted 16S rRNA gene sequencing of lung wash samples obtained from 14 infant monkeys (*H. pylori* positive n = 6; *H. pylori* negative n = 8) which were a subset of our original 25 animal cohort (Supplemental Table 1). Buccal swabs were also obtained from 12 out of the 14 animals prior to lung washes to compare microbiome composition between the oral and lung mucosal surfaces. A median number of 23,145 reads were generated per lung wash sample (range = 10,693–65,361 reads) and a median number of 56,690 reads were generated per buccal swab sample (range = 36,991–80,043 reads).

**Figure 1:**
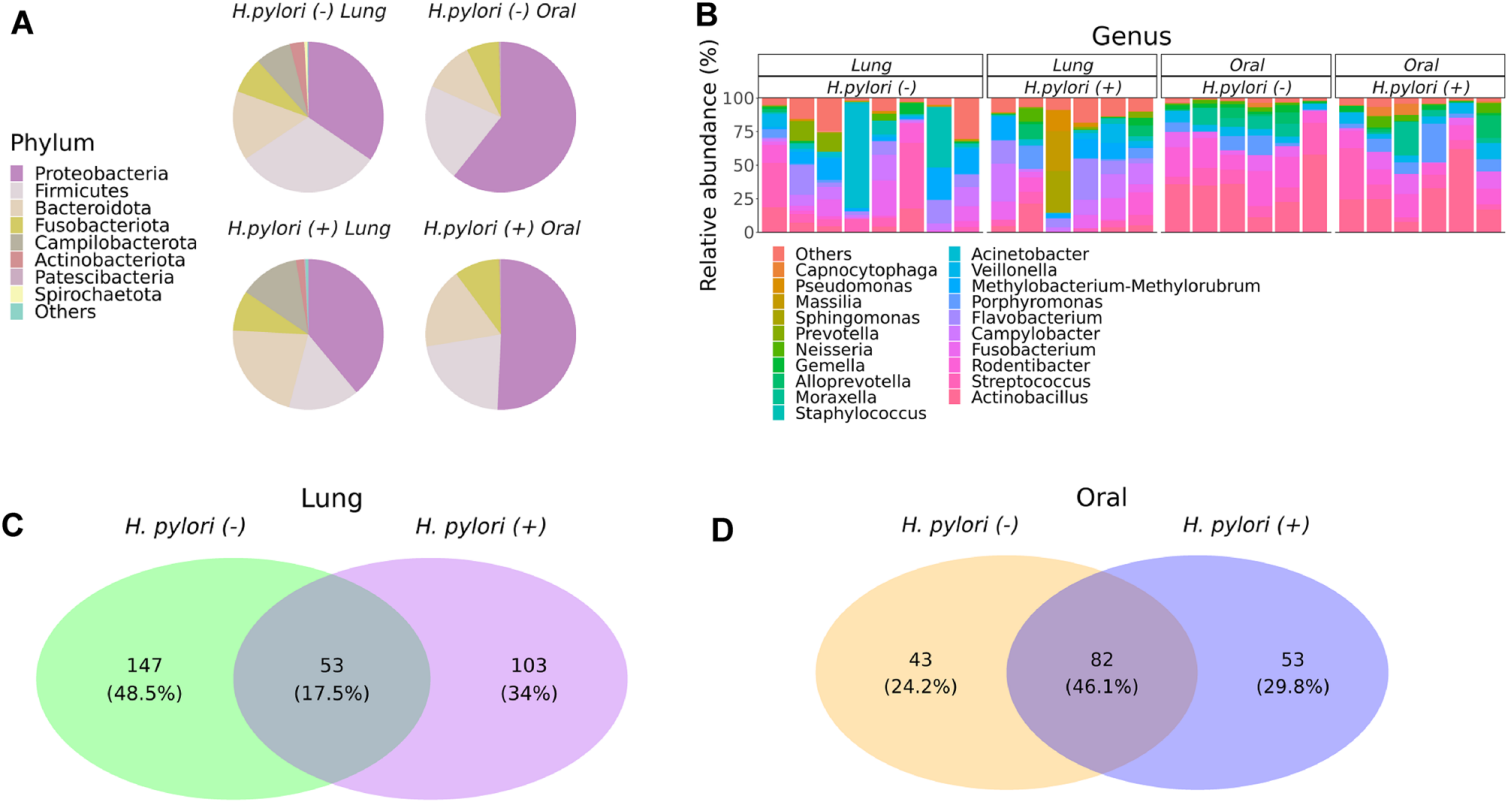
The composition of the infant monkey lung and oral microbiomes are distinct. (**A**) Pie chart for average abundance of phyla in the lung (n = 6 *H. pylori* + , n = 8 *H. pylori* -) and oral (n = 6 *H. pylori* + , n = 6 *H. pylori* -) samples from infant monkeys, separated by *H. pylori* infection status. (**B**) Relative abundance of microbiota for each animal at the genus level for lung and oral samples, separated by *H. pylori* infection status. (**C**) Venn diagram of the lung microbiome for infant monkeys separated by gastric *H. pylori* infection status, representing shared and exclusive genus-level taxa. (**D**) Venn diagram of the oral microbiome for infant monkeys separated by *H. pylori* infection status, representing shared and exclusive genus-level taxa.

The lung microbiome of infant monkeys for both *H. pylori* positive and negative animals was primarily composed of the phyla (in order of relative abundance) Proteobacteria, Firmicutes, Bacteroidota, Fusobacteriota, Campilobacterota and Actinobacteriota (Fig. 1(A)). The oral microbiome for infant monkeys was primarily composed of the phyla (in order of relative abundance) Proteobacteria, Firmicutes, Bacteroidota, and Fusobacteriota. At the genus level, *Actinobacillus*, *Streptococcus*, *Rodentibacter*, *Fusobacterium*, *Porphyromonas*, *Campylobacter*, *Flavobacterium*, and *Veillonella* were most abundant in the lung microbiome, while *Actinobacillus*, *Streptococcus*, *Rodentibacter*, *Fusobacterium*, *Porphyromonas*, and *Veillonella* were most abundant in the oral microbiome (Figs. 1(B), S3). The lung microbiome of *H. pylori* infection positive and negative infant monkeys shared 17.5% of genus-level taxa (Fig. 1(C)). The oral microbiome was less affected by *H. pylori* infection status, with 44.9% of genus-level taxa shared between *H. pylori* positive and negative infant monkeys (Fig. 1(D)). We were unable to find *H. pylori* in either the lung or oral cavity samples despite gastric infection; however, we did detect *H. fennelliae* in the lavage of 2 gastric *H. pylori*-negative animals (data not shown).

### Effect of* H. pylori* infection on the infant monkey lung and oral microbiome

**Figure 2:**
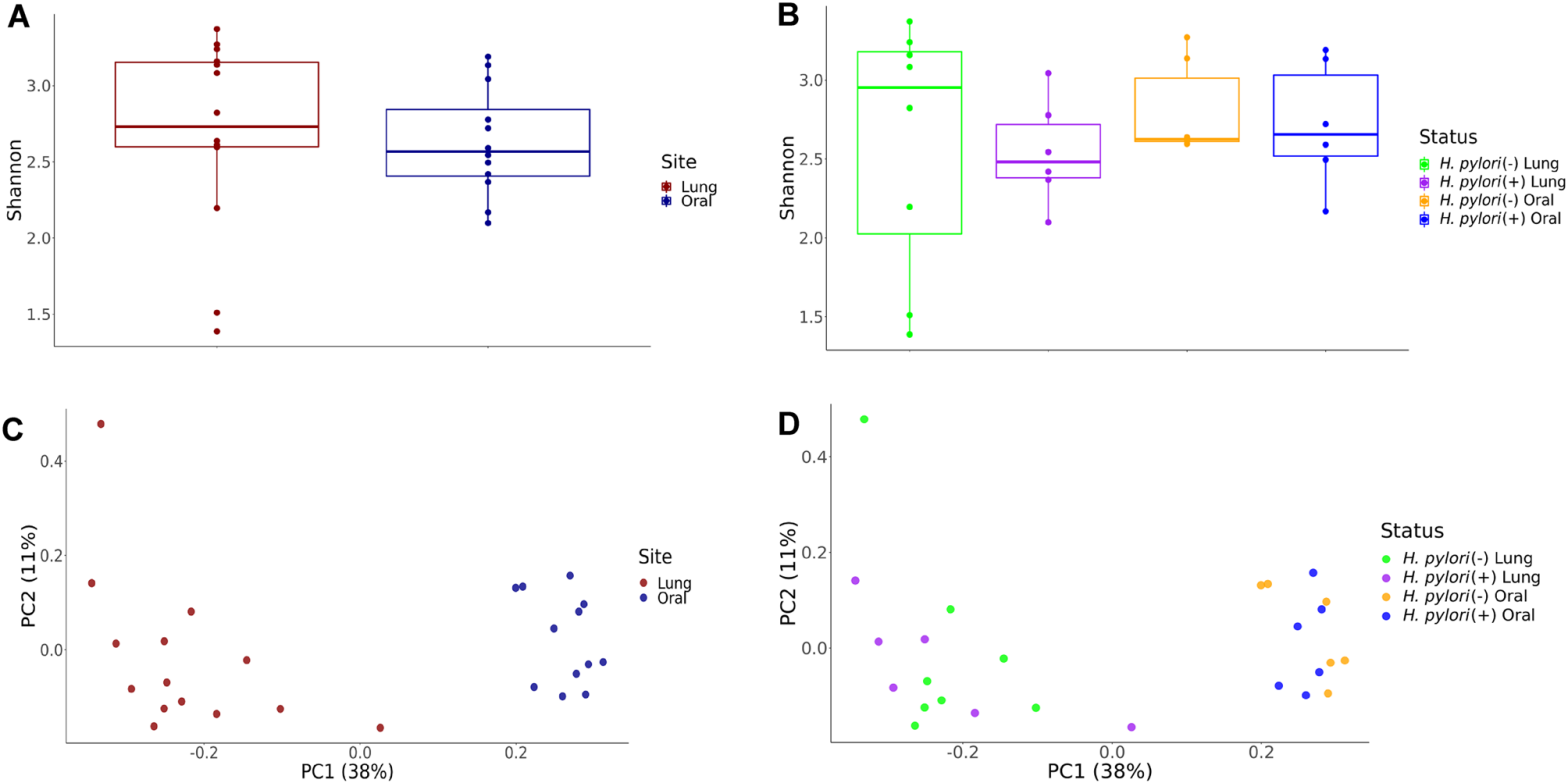
Diversity of the infant monkey lung and oral microbiome. (**A**) Shannon index of lung microbiome separated by gastric *H. pylori* infection status. (**B**) Shannon index of oral microbiome separated by gastric *H. pylori* infection status. (**C**) Principal component analysis of evenness (unweighted UniFrac distance) separated by lung versus oral microbiome. (**D**) Principal component analysis of evenness (unweighted UniFrac distance) separated by lung versus oral microbiome and gastric *H. pylori* infection status.

To assess the impact of *H. pylori* infection on the composition of the lung and oral microbial communities in infant monkeys, we examined the alpha and beta diversity metrics between *H. pylori* positive versus negative animals. The Shannon diversity index did not significantly differ between the lung and oral samples as well as relative to *H. pylori* infection status in our study animals (Fig. 2(A,B)). Principal component analysis (PCA) of unweighted UniFrac distance showed distinct lung and oral microbiomes that did not cluster by *H. pylori* infection status (Fig. 2(C,D)).

**Figure 3:**
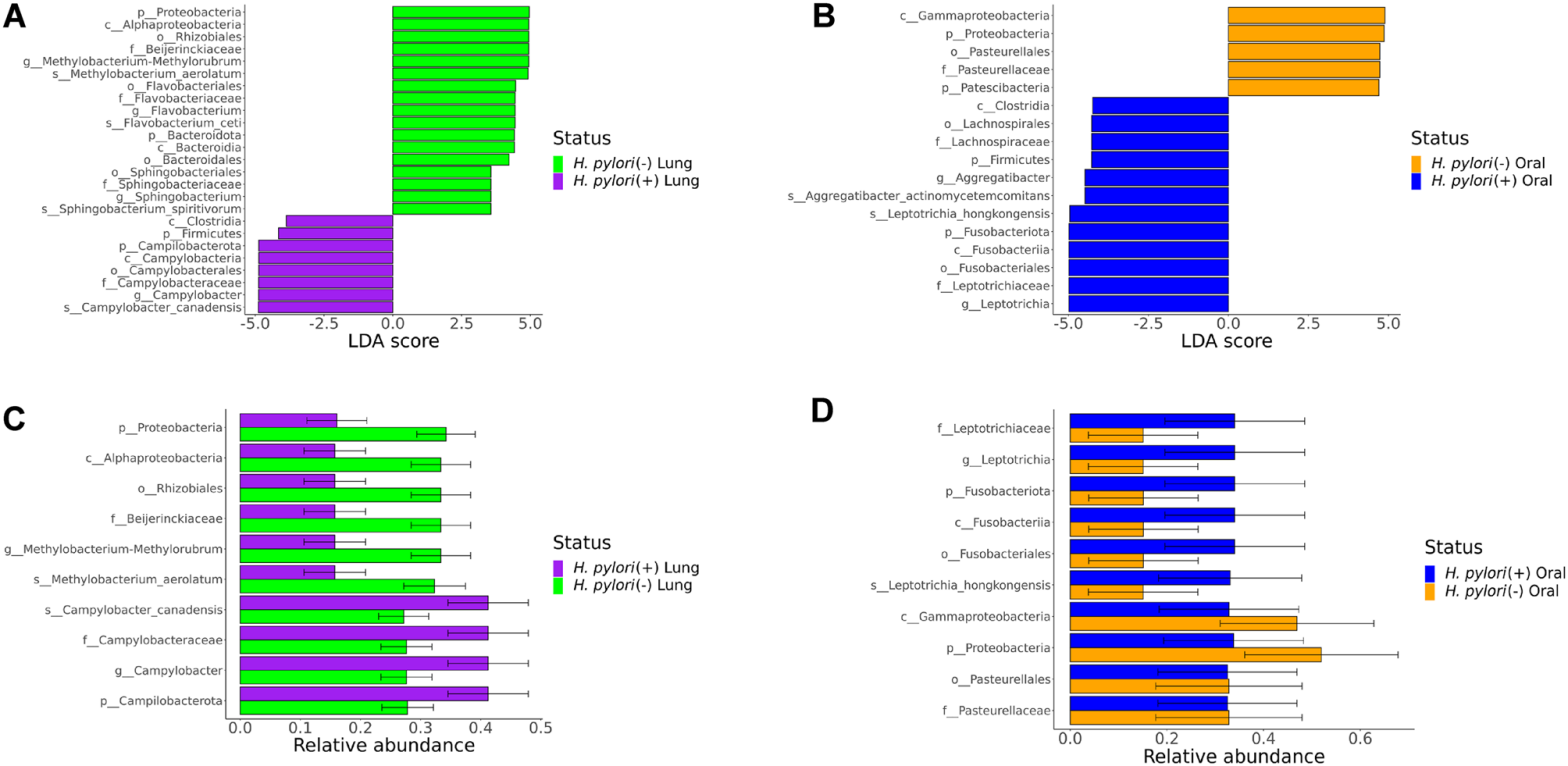
The lung and oral microbiomes of infant monkeys are composed of different taxa. (**A**) Differential abundance in the lung microbiome relative to gastric *H. pylori* infection status was assessed using LEfSe. (**B**) Differential abundance in the oral microbiome relative to gastric *H. pylori* infection status was assessed using LEfSe. (**C**) Relative abundance (% of total reads) of top taxa in the lung microbiome identified in LEfSe analysis. (**D**) Relative abundance (% of total reads) of top taxa in the lung microbiome identified in LEfSe analysis. (alpha value = 0.05, logarithmic LDA score threshold = 2, error bars represent SEM).

The lung was composed of more genera and species that significantly differed by *H. pylori* status than the oral cavity. A total of 6 genera and species were significantly increased in lung washes from *H. pylori* negative infant monkeys relative to *H. pylori* positive animals, including *Methylobacterium*-*Methylorubrum* (LDA = 4.9349, *p* = 0.0001), *Methylobacterium aerolatum* (LDA = 4.9058, *p* = 0.0001)*, Flavobacterium* (LDA = 4.5634, *p* = 0.0001), *Flavobacterium ceti* (LDA = 4.5634, *p* = 0.0001), *Sphingobacterium* (LDA = 3.4742, *p* = 0.0059), *Sphingobacterium spiritivorum* (LDA = 3.4742, *p* = 0.0059) (Fig. 3(A)). The one genus and species increased in lung wash from *H. pylori* positive infant monkeys relative to *H. pylori* negative animals was *Campylobacter* (LDA = 4.8207, *p* = 0.0001) and *Campylobacter canadensis* (LDA = 4.8365, *p* = 0.0001). Genera and species that were significantly increased from buccal swabs of *H. pylori* positive versus negative infant monkeys were *Aggregatibacter* (LDA = 4.4085, *p* = 0.0039), *Aggregatibacter actinomycetemcomitans* (LDA = 4.4035, *p* = 0.0039)*, Leptotrichia* (LDA = 4.9752, *p* = 0.0039), and *Leptotrichia hongkongensis* (LDA = 4.9317, *p* = 0.0059) (Fig. 3(B)). Buccal swabs from *H. pylori* negative infant monkeys had increased p__Proteobacteria (LDA = 5.0132, *p* = 0.0005), c__ Gammaproteobacteria (LDA = 4.9679, *p* = 0.0039), o__Pasteurellales (LDA = 4.6916, *p* = 0.0039), and p__ Patescibacteria (LDA = 4.5968, *p* = 0.0092) relative to *H. pylori* positive animals, but no differences at the genus or species level. Relative abundance of taxa identified through LEfSe revealed no overlap between the top 10 taxa in the lung and oral microbiomes, except for p Proteobacteria, which was enriched in *H. pylori* negative infant monkeys (Fig. 3(C,D)). The relative abundance of 6 of the top 10 taxa in the lung microbiome of *H. pylori* negative infants tended to be higher in comparison with *H. pylori* positive animals. Conversely, the relative abundance of 6 of the top 10 taxa in the oral microbiome tended to be higher in *H. pylori* positive infant monkeys in comparison with *H. pylori* negative animals.

### Infant monkey lung IL-8 is associated with* H. pylori* gastric load and microbial composition

**Figure 4:**
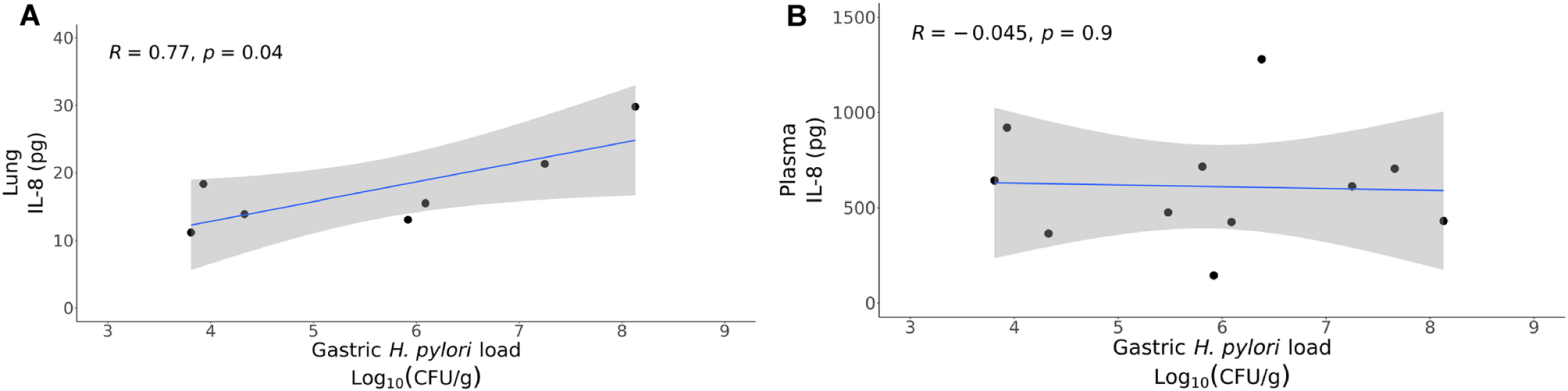
*H. pylori* infection status correlates with increased IL-8 in the lung. (**A**) Linear fit of gastric *H. pylori* load and lung wash IL8 concentration (*p* = 0.04, R = 0.59). (**B**) Linear fit of gastric H. pylori load and plasma IL8 concentration (*p* = 0.89, R = 0.002).

Increased IL-8 protein in the gastric mucosa has been detected in children infected with *H. pylori* relative to healthy subjects, although circulating IL-8 levels may be reduced^36,37^. To assess whether *H. pylori* colonization during rhesus monkey postnatal development is similarly associated with elevated IL-8 expression at other mucosal or systemic sites, we measured IL-8 protein in lung wash and plasma samples collected in conjunction with microbiome sampling. *H. pylori* gastric load significantly correlated with lung wash IL-8 protein concentration (r = 0.59, *p* = 0.04), although we did not detect a significant difference when just comparing *H. pylori* infection status (negative versus positive) (Figs. 4(A), S4A). In contrast with lung washes, we did not observe an association between plasma IL-8 concentration and *H. pylori* gastric load or infection status (Figs. 4(B), S4B). Plasma but not lung IL-8 concentration did correlate with the chronologic age of animals (*p* = 0.02, R = 0.22, Fig. S5), but there was no correlation of plasma IL-8, age and H. pylori status in a mixed effects model (Fig. S6, Supplemental Table 2). There were no sex differences in lung or plasma IL-8 concentration for infant monkeys in this study (Figs. S7A,B).

**Figure 5:**
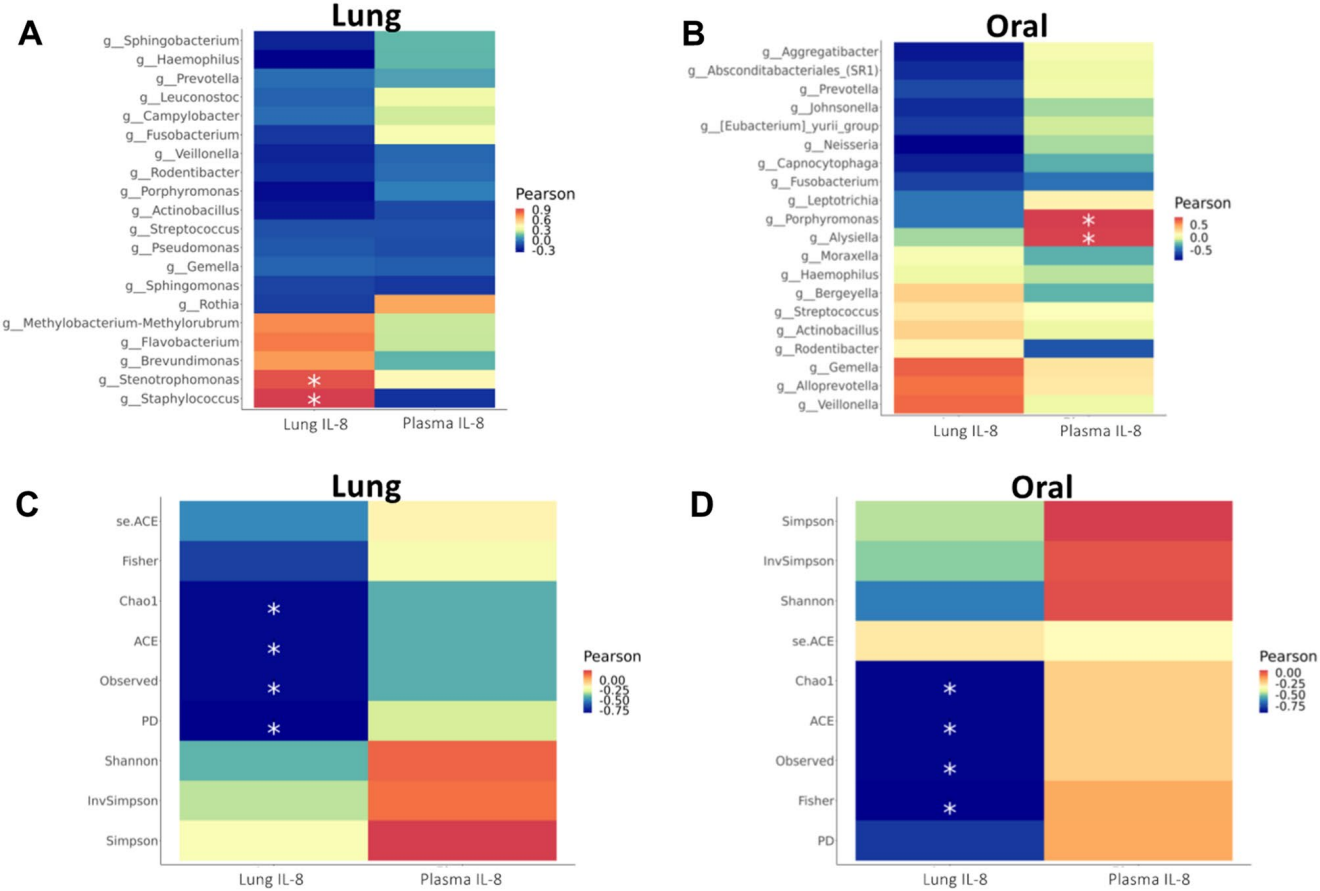
Microbial genera influence IL-8 in the lung and plasma independent of *H. pylori* status. (**A**) Heatmaps of Pearson correlation coefficients were generated for abundance of lung microbial genera versus IL-8 concentration in the lung or plasma. (**B**) Heatmaps of Pearson correlation coefficients were generated for abundance of oral microbial genera versus IL-8 concentration in the lung or plasma. (**C**) Heatmaps of Pearson correlation coefficients were generated for lung microbiome alpha diversity metrics versus IL-8 concentration in the lung or plasma. (**D**) Heatmaps of Pearson correlation coefficients were generated for oral microbiome alpha diversity metrics versus IL-8 concentration in the lung or plasma. Red color indicates a positive correlation coefficient, and blue color represents a negative coefficient. Significant correlations (adjusted *P* value < 0.05) are indicated with an asterisk.

To determine whether there was a relationship between *H. pylori* infection, relative abundance of genera and IL-8 concentration, we grouped samples by location(lung versus oral) and *H. pylori* status. There were no significant correlations between *H. pylori* positive versus negative animals in the lung (Fig. S8A) or oral microbiome (Fig. S8B) in association with microbial genera abundance and IL-8 concentration. We next sought to assess the relationship between IL-8 production and the most abundant bacteria in the lung and oral microbiota independent of gastric *H. pylori* status. *Staphylococcus* was one of the most abundant taxa in the lung wash microbiome and was positively correlated with lung IL-8 (r = 0.93, *p* = 0.02) but not plasma IL-8 (Fig. 5(A)). Additionally, *Stenotrophomonas* positively correlated with lung IL-8 (r = 0.88, *p* = 0.04). Conversely, no genera from the buccal swab correlated with lung IL-8. *Porphyromonas* (r = 0.78, *p* = 0.047) and *Alysiella* (r = 0.78, *p* = 0.047) positively correlated with plasma IL-8 (Fig. 5(B)). Lastly, we interrogated whether alpha diversity indices correlated with IL-8 protein concentration, including community richness (observed species, Chao1 and ACE), community diversity (Shannon, Simpson), and phylogenetic diversity. We found diversity measures including Chao1, ACE, Observed ASVs, Fisher, and phylogenetic diversity negatively correlated with lung but not plasma IL-8 for both the lung and oral microbiomes (Fig. 5(C,D)).

## Discussion

Because the human intestinal microbiome is known to display a temporal progression toward greater diversity and stability during the first 2–3 years of life, we hypothesized that *H. pylori* infection during infancy might alter the composition of commensal populations at mucosal sites distant from the gastric region. Using the infant rhesus macaque monkey to model neonatal development, we first profiled the lung and oral microbiome by 16S rRNA gene sequencing in a cohort of animals that were 6–7 months of age. We subsequently determined the relationship of the infant monkey lung and oral microbiome relative to gastric *H. pylori* infection status as well as IL-8 protein expression. Independent of gastric *H. pylori* colonization, we found the lung microbiome of infant monkeys was dominated by the phyla Proteobacteria, Firmicutes, Bacteroidota, Fusobacteriota, Campilobacterota and Actinobacteriota, which was distinct from the oral microbiome that was dominated by Proteobacteria, Firmicutes, Bacteroidota, and Fusobacteriota. In the lung, *H. pylori* infection status was associated with shifts in the abundance of seven genera and species. Comparatively, there were fewer genera and species for the oral cavity relative to *H. pylori* infection status. Because increased gastric IL-8 expression has been reported to be associated with *H. pylori* colonization in human adults and children, we assessed whether airway or systemic IL-8 levels might a) reflect *H. pylori* infection status and b) correlate with microbial communities withing the lung or oral cavity^36,38^. We found the lung, but not plasma IL-8 protein concentration associated with gastric *H. pylori* load, differences in the genera *Stenotrophomonas* and *Staphyloccus*, and microbial diversity for both lung and oral cavities. Plasma IL-8 protein concentration was only associated with differences in the genera *Alysiella* and *Moraxella* in the oral cavity.

Our findings on the commensal microbiome detected in lung wash from infant monkeys are similar to reports of 16S rRNA gene sequencing conducted on lung lavage collected from adult cynomolgus or rhesus macaques. In a longitudinal assessment of lavage obtained by bronchoscopy from cynomolgus macaques infected with SIV-HIV, the phyla Proteobacteria, Bacteroidota, Fusobacteriota, Firmicutes, and Actinobacteriota were predominant^39^. Consistent with findings in adult cynomolgus macaques, a study of aging rhesus macaque monkeys also described predominating in lung lavage the phyla Proteobacteria, Bacteroidota, Fusobacteriota, Firmicutes, and Actinobacteriota^40^. At the genus level, a comparison of our data indicates a substantial overlap between infant and adult rhesus monkey lung microbial communities, with a prevalence of *Actinobacillus*, *Streptococcus*, *Rodentibacter*, *Fusobacterium*, *Porphyromonas, Veillonella*, *Staphylococcus*, *Moraxella*, *Gemella*, *Neisseria*, and *Prevotella* for both age groups^40^. A distinctive finding of prior lung lavage studies in adult cynomolgus and rhesus monkeys is the dominant contribution of *Tropheryma whipplei* in subsets of characterized animals, ranging from one fourth to two thirds of surveyed cohorts^39,40^. *Tropheryma whipplei* has been observed in the respiratory tract of HIV infected subjects, but the basis for colonization is unknown^41^. *Tropheryma whipplei* was not reported in a separate study of 26 adult cynomolgus macaque monkeys, therefore colonization with this species may vary by animal source^42^. In our study of the infant rhesus monkey lung, we did not observe *Tropheryma whipplei* as a major constituent of the microbiome, however it is possible the use of a sterile endotracheal tube to collect a lung wash may not have captured a species that is located within more distal alveoli. Lavage from adult macaque monkeys also did not appear to contain the phyla Campilobacterota, which was found in lung wash from infant macaque monkeys in our study, but the genus *Campylobacter* has been reported in the lung of cynomolgus macaque monkeys following infection with Mycobacterium tuberculosis^40,42^. Interestingly, Campylobacter in the human lung is associated with inflammatory and immunocompromised conditions such as chronic obstructive pulmonary disease^43,44^. It is notable that *Campylobacter* was only detected in the lung but not oral cavity of animals in our study, suggesting an alternative anatomical route (e.g. nasal cavity) into the airways.

For the oral microbiome, Proteobacteria was the predominant phylum observed for infant rhesus monkeys, however we have previously found the phylum Firmicutes as the most predominant source of reads from the oral cavity of adult rhesus monkeys from the same breeding colony as our current study^45^. Chen, et al. reported the composition of the oral microbiome from a population of wild adult macaque monkeys consisting of rhesus (*M. mulatta*) and cynomolgus (*M. fascicularis*) hybrid monkeys closely resembled captive adult rhesus monkeys and adult humans, with Firmicutes followed by Proteobacteria and Bacteroidota as the most abundant phyla^46^. Differences in relative abundance of phyla may be due to inter-animal variability, however the consistency of oral microbial communities from both captive and wild macaque monkeys suggests that chronological age of the infant monkeys in our study is a contributing factor to the observed microbial signature of the lung or oral cavity. Indeed, there is evidence from longitudinal assessment of saliva from humans starting at the first week of life that fluctuations in abundance and composition of the oral microbiome continues until five years of age^47^. The oral microbiome of 18-month-old children significantly differs from their respective parents, which lends further support to the notion that adult microbial communities are progressively shaped by environmental exposures^48,49^.

We sought to assess the impact of *H. pylori* infection on the lung and oral microbiome of the infant monkey due to its inverse association with asthma development in childhood. Inflammatory mediators such as IL-8 have been shown to increase with *H. pylori* infection both in plasma and the gastric mucosa^37,50,51^. When characterizing the IL-8 profiles of lung wash and plasma from the study cohort of 25 infant rhesus macaques, we found that lung, but not plasma, correlated with gastric *H. pylori* load. Yet while lung lavage IL-8 levels positively correlated with *H. pylori* load, we were unable to detect *H. pylori* in the infant lung or oral samples collected for this study. Microbes aspirated from the oral cavity are subject to rapid mucociliary clearance, therefore detection may be challenging. Our prior in vitro studies have demonstrated that *H. pylori* infection can directly elicit IL-8 secretion in rhesus monkey airway epithelium in an age-dependent manner, with a greater than four-fold induction in infant versus adult-derived cultures^52^. As such, it is feasible that even brief exposure to *H. pylori* in the respiratory tract without colonization could be a mechanism for the increased IL-8 observed in lung wash samples from infant monkeys in our study. Alternatively, IL-8 may be sourced from trafficking immune cells induced by *H. pylori*, which is one proposed mechanism by which the gastrointestinal tract can communicate with distal sites such as the lung, and similar to the induction of T regulatory cells in a murine model of allergic asthma^24,25,53^.

Source communities for the adult lung microbiome are likely to be organisms from the oral cavity and the upper respiratory tract but the oral microbiome is distinct from the lung microbiome^54,55^. Comparatively, we did not detect statistically significant differences in alpha diversity measures between the lung and oral microbiome of infant monkeys in our study, but beta diversity measures showed distinct clustering of lung and oral microbiomes. In the lung, we found a significant positive correlation between lung IL-8 and the genera *Staphylococcus* and *Stenotrophomonas* while there was a significant positive correlation between plasma IL-8 and the genera *Porphyromonas* and *Alysiella* in the oral cavity. Given that *Staphylococcus aureus* and *Stenotrophomonas maltophilia* are opportunistic pathogens in the respiratory tract and frequently detected in cystic fibrosis patients, it may be speculated that innate immune function in the lung mucosa could contribute to shifts in these genera^56^. The pathogenicity of *Alysiella* is unknown but it is commonly found in the oral cavity of mammals, whereas *Porphyromonas gingivalis* is associated with periodontal disease^57,58^. Our finding of the compartmental source of IL-8 differentially correlated with genera within anatomical sites supports the notion of that mucosal microenvironments distinctly contribute to the microbial milieu.

To date, investigation of the respiratory microbiome in pediatric populations have focused on minimally invasive sampling sites, such as the nasopharynx. The composition of the nasopharyngeal microbiome has been associated with respiratory infections and asthma development^50^. However, nasopharyngeal microbial communities are significantly different from the actual lung microbiome in adulthood and tend to be similar to the microbial composition of the skin^19,55^. An alternative approach to assess the pediatric respiratory microbiome is voluntary sample production, such as sputum. Sputum analysis as permitted comparison of lung microbiomes from pediatric and adult cystic fibrosis patients, which detected few differences in core lung microbiota in association with age^59^. It can be difficult to interpret sputum samples for lung microbial communities as this type of collection runs the risk of upper respiratory contamination and significant contamination from the oral cavity^60^. A higher abundance of Proteobacteria in adult and pediatric asthma patients relative to control subjects has been observed in bronchoalveolar lavage, however the study was limited by variability in age, sex, and disease status^17^. Due to technical and ethical challenges of an invasive sampling approach, the lung microbiome of healthy human infants is a substantial knowledge gap that is crucial for addressing causality 16S rRNA sequencing data collected during disease states in pediatric populations.

While the findings from this study address an important knowledge gap for development of the infant lung microbiome in a primate species, there are several limitations of the study to consider. Most notably, the small sample size which may have reduced our ability to detect differences in alpha diversity based upon *H. pylori* status or load. Another potential limitation of this study is that it was not possible to discern the disease state of animals; gastric cultures may provide some evidence of the state of colonization but children and rhesus macaques have been found to spontaneously clear infection^61,62^. We also detected an inverse relationship between infant *H. pylori* infection and maternal age, which may have influenced the microbial status of offspring; the microbial milieu of younger dams may differ from older dams. Indeed, there is a positive association of age with alpha diversity in the human gut microbiome that plateaus after 40 years of age^63^. Additionally, while we found no evidence that housing conditions contributed to the observed differences in the infant lung and oral microbiome with *H. pylori* infection, it is feasible that sampling a larger cohort would detect such environment-associated effects.

In summary, we assessed the impact of an early life gastric infection on the infant lung and oral microbiome by taking advantage of an endemic *H. pylori* colonization in a captive cohort of infant rhesus macaque monkeys. To the best of our knowledge, this is the first study to characterize the normal infant lung microbiome in a primate species. Moreover, our data suggests the postnatal primate lung harbors a microbiome signature that is distinct from the oral cavity. Gastric *H. pylori* colonization was associated with a shift in the composition of microbial communities in the lung and oral cavity; future studies would be needed to determine if an altered microbiome persists as *H. pylori* infected animals mature into adulthood. The therapeutic value of *H. pylori*-derived components for reducing allergic airways inflammation is under investigation; given our present findings it may be of interest to assess whether altering the lung microbiome during infancy could prevent development of childhood asthma^64,65^.

### Supplementary Information

Supplementary Information.

## Data Availability

Microbiome 16S sequencing data are available under the accession number PRJNA773421. Analysis codes are available at https://github.com/nasiegel88/H.pylori-siegel_et_al_2022.
