# Supplementary material for: Helicobacter pylori infection in infant rhesus macaque monkeys is associated with an altered lung and oral microbiome
Source: Sci Rep. 2024 May 1;14:9998. doi: 10.1038/s41598-024-59514-5 (PMC11063185; doi:10.1038/s41598-024-59514-5)
Supplement: Supplementary file 1 — Supplementary Information. [file 41598_2024_59514_MOESM1_ESM.docx]

# ***Helicobacter pylori* Infection in Infant Rhesus Macaque Monkeys is Associated with an Altered Lung and Oral Microbiome**

Noah A. Siegel^1^, Monica T. Jimenez^1^, Clarissa Santos Rocha^2^, Matthew Rolston^2^, Satya Dandekar^1,2^, Jay V. Solnick^1,2^ and Lisa A. Miller^1,3*^

^1^California National Primate Research Center, University of California Davis, Davis, California, United States of America

^2^Department of Medical Microbiology and Immunology, School of Medicine, University of California Davis, Davis, California, United States of America

^3^Department of Anatomy, Physiology and Cell Biology, School of Veterinary Medicine, University of California Davis, Davis, California, United States of America

*Correspondence: lmiller@ucdavis.edu

NAS, MTJ, and CSR are Joint First Authors

LM and JV are Joint Senior Authors

**Supplementary Tables and Figures**

**Supplemental Fig 1** *H. pylori* infection status was not associated with the chronologic age of study animals. **A** Age of all gastric *H. pylori* positive versus negative infant monkeys in this study. **B** Age of gastric *H. pylori* positive versus negative infant monkeys in this study evaluated by 16s sequencing. Significant differences between groups were assessed using non-parametric t-tests (P-value <0.05).

**Supplemental Fig 2** *H. pylori* gastric load in infant monkeys is associated with the chronologic age of dams. Linear fit of infant monkey gastric *H. pylori* load and dam age in months (p=0.007, R=-0.58).

**Supplemental Fig 3** Pie chart for average abundance at the genus level in the lung (n=6 *H. pylori* +, n=8 *H. pylori* -) and oral (n=6 *H. pylori* +, n=6 *H. pylori* -) samples from infant monkeys, separated by *H. pylori* infection status

**Supplemental Fig 4** *H. pylori* infection status was not associated with lung or plasma IL-8 concentration in study animals. **A** Lung IL-8 concentration in gastric *H. pylori* positive versus negative infant monkeys in this study (p=0.46). **B** Plasma IL-8 concentration in gastric *H. pylori* positive versus negative infant monkeys in this study (p=0.79). Significant differences between groups were assessed using non-parametric t-tests (P-value <0.05).

**Supplemental Fig 5** Chronologic age of study animals was associated with plasma IL-8 concentration. Linear fit of plasma IL-8 concentration and chronologic age (days) for both gastric *H. pylori* positive and negative infant monkeys in this study (p=0.02, R=0.22).

**Supplemental Fig 6** *H. pylori* status in a linear mixed-effects model of plasma IL8 concentration over age (measured in days). Lines are depicted with a 95% confidence interval.

**Supplemental Fig 7** Biological sex of study animals was not associated with lung or plasma IL-8 concentration. **A** Lung IL-8 concentration in female versus male infant monkeys in this study (p=0.53). **B** Plasma IL-8 concentration in female versus male infant monkeys in this study (p=0.95). Significant differences between groups were assessed using non-parametric t-tests (P-value <0.05).

**Supplemental Fig 8** Association of *H. pylori* status with microbial genera abundance and IL-8 in the lung and plasma. Heatmaps of Spearman correlation coefficients were generated for abundance of microbial genera and IL-8 concentration in the **(A)** lung and **(B)** plasma. The red color indicates a positive correlation coefficient, and the blue color represents a negative coefficient. Significant correlations (adjusted P-value <0.05) were indicated with an asterisk.

**Supplemental Table I** Demographics and housing for study animals evaluated by 16S rRNA sequencing (n=14)

| Animal | Sex | Infant age  (months) | Weight  (kg) | *H. pylori*  status | *H. pylori* load  (log10 cfu/g tissue) | Dam age  (months) | Housing  status |
| --- | --- | --- | --- | --- | --- | --- | --- |
| 1 | Female | 6 | 1.54 | - | 0 | 103 | Room 2 |
| 2 | Female | 6 | 1.37 | - | 0 | 76.6 | Room 2 |
| 3 | Female | 6.1 | 1.69 | - | 0 | 118.8 | Room 4 |
| 4 | Female | 6.2 | 1.4 | - | 0 | 91 | Room 2 |
| 5 | Female | 6.4 | 1.3 | - | 0 | 90.4 | Room 2 |
| 6 | Female | 6.6 | 1.2 | + | 7.66 | 69.3 | Room 2 |
| 7 | Female | 6.8 | 1.5 | + | 6.09 | 80.1 | Room 2 |
| 8 | Female | 7 | 1.4 | + | 3.81 | 104.8 | Room 2 |
| 9 | Male | 7.1 | 1.54 | + | 5.48 | 115 | Room 1 |
| 10 | Female | 7.2 | 1.3 | - | 0 | 102.9 | Room 3 |
| 11 | Female | 7.3 | 1.44 | - | 0 | 106.9 | Room 4 |
| 12 | Female | 7.6 | 1.73 | - | 0 | 117.1 | Room 2 |
| 13 | Female | 7.7 | 1.87 | + | 5.81 | 117.9 | Room 2 |
| 14 | Female | 7.8 | 1.64 | + | 6.38 | 93.4 | Room 4 |

**Supplemental Table 2** Plasma IL-8 concentration linear mixed effects model; variance of fixed effects was assessed through ANOVA

|  | **Sum Sq** | **Mean Sq** | **NumDF** | **DenDF** | **F value** | **Pr(>F)** |
| --- | --- | --- | --- | --- | --- | --- |
| Age (days) | 306252.3 | 306252.3 | 1 | 19 | 4.694 | 0.043 |
| H. pylori Status | 8304.796 | 8304.796 | 1 | 19 | 0.127 | 0.725 |
| Age (days): H. pylori Status | 12615.25 | 12615.25 | 1 | 19 | 0.193 | 0.665 |
